# A systematic review of the prevalence of psychosis in people with tuberculosis

**DOI:** 10.1192/j.eurpsy.2024.759

**Published:** 2024-08-27

**Authors:** S. M. Gunawardena, E. McMahon, A. M. Doherty

**Affiliations:** ^1^Department of Psychiatry, University College Dublin, 63 Eccles Street; ^2^Department of Liaison Psychiatry, The Mater Misericordiae University, Dublin 7, Ireland

## Abstract

**Introduction:**

Tuberculosis is a bacterial infectious disease caused by Mycobacterium tuberculosis. This disease predominately affects the lungs but also affects other parts of the body, including the central nervous system. According to the the World Health Organization (2023), tuberculosis has an incidence of 6.4 million people in 2022, with 1.5 million deaths attributed to this disease. Psychosis describes a group of disorders that affects a person’s thought process and perception. It is a serious disorder that can have a profound impact on a person’s mental and physical health. As a result, psychosis symptoms and its treatment can complicate the management of tuberculosis.

**Objectives:**

The aim of this systematic review is to explore the association between tuberculosis and psychosis. It has been shown that up to 70% of patients with tuberculosis also have comorbid mental illness, this is likely to include psychosis. There are also shared risk factors between tuberculosis and psychosis, including poverty and homelessness, substance abuse, HIV positive serology and isolation. Tuberculosis medication, including isoniazid and rifampicin have been shown to have adverse psychiatric effects and we will examine if this includes psychosis.

**Methods:**

A systematic review was pre-registered with PROSPRO and performed using Preferred Reporting Items for Systematic Reviews and Meta-Analyses guidelines. MEDLINE, OVID and PsychINFO databases were searched from beginning of records to September 2023. This included hand-search of relevant reference lists. Observational and epidemiological studies were included along with population based registries.

**Results:**

Over one thousand (1,154) articles were identified and screened. There was significant heterogenity in results and over half of studies were from Asia and Africa. Many studies reported cases of drug-induced psychosis from anti-tubercular agents. Studies also discussed the increased risk of TB incidence among patients with psychosis and other psychiatric disorders.

**Conclusions:**

This study identifies the importance of training healthcare workers in rapid detection of co-morbid psychosis in patients with tuberculosis, along with neuropsychiatric side effects of antitubercular agents. Integration of psychiatric and medical care of these patients would be of benefit to improve outcomes in this patient population. More research is needed on co-morbidity of tuberculosis and psychosis.

**Disclosure of Interest:**

None Declared

